# Direction of association between Cardiovascular risk and depressive symptoms during the first 18 years of life: A prospective birth cohort study

**DOI:** 10.1016/j.jad.2021.05.094

**Published:** 2021-09-01

**Authors:** Anna B. Chaplin, Nick Smith, Peter B. Jones, Golam M. Khandaker

**Affiliations:** a Department of Psychiatry, University of Cambridge, Cambridge, United Kingdom; b Cambridgeshire and Peterborough NHS Foundation Trust, Cambridge, United Kingdom; c James Paget University Hospital, Norfolk, United Kingdom; d MRC Integrative Epidemiology Unit, Population Health Sciences, Bristol Medical School, University of Bristol, Bristol, United Kingdom; e Centre for Academic Mental Health, Population Health Sciences, Bristol Medical School, University of Bristol, Bristol, United Kingdom; f Avon and Wiltshire Mental Health Partnership NHS Trust, Bristol, United Kingdom

**Keywords:** ALSPAC, Longitudinal study, Depressive symptoms, Cardiovascular risk, Adolescence, Inflammation

## Abstract

**Background:**

Cardiovascular disease (CVD) and depression are bidirectionally associated in adults. However, the direction of association between CVD risk and depressive symptoms in young people and potential mechanisms are poorly understood.

**Methods:**

Using longitudinal birth cohort data, we created a CVD risk score age at 15 using age, ethnicity, physical activity, maternal social status, maternal smoking, own smoking, BMI, systolic blood pressure, LDL, HDL and triglycerides. We used regression analysis to test: (1) association between CVD risk score at age 15 and depressive symptoms at ages 12 and 18; (2) association of IL-6 and CRP at age 9 with depressive symptoms at age 12 and CVD risk score at age 15; and (3) mediating effects of CVD risk score on associations of IL-6/CRP at age 9 with depressive symptoms at age 18.

**Results:**

The risk set comprised 5007 participants. CVD risk score in mid-adolescence was associated with depressive symptoms in early-adulthood (adjusted beta=0.06; standard error (SE)=0.02; *p*<0.001). Depressive symptoms in childhood were not associated with CVD risk score in mid-adolescence (adjusted beta=0.03; SE=0.02; *p*=0.11). Childhood inflammatory markers were associated with CVD risk score in mid-adolescence. Adolescent CVD risk score mediated the associations between childhood inflammatory markers and depressive symptoms in early-adulthood.

**Limitations:**

The cohort primarily comprises White individuals, limiting generalisability. Sample attrition required imputation for missing data.

**Conclusions:**

Association between CVD risk and depression in childhood/adolescence is unidirectional, with higher CVD risk increasing the risk of depressive symptoms. Childhood inflammation may increase risk of depression by influencing adolescent CVD risk.

## Highlights

• Higher cardiovascular risk score at age 15 is associated with depressive symptoms at age 18.
• Depressive symptoms at age 12 are not associated with cardiovascular risk score at age 15.
• Association between depression and cardiovascular risk in young people may be unidirectional.
• IL-6 and CRP levels at age 9 are associated with cardiovascular risk score at age 15.
• Adolescent cardiovascular risk mediates the association between childhood inflammation and early-adult depressive symptoms.

## 1 Introduction

Depression and cardiovascular disease (CVD) commonly co-occur and are bidirectionally associated in adults. Depression is associated with increased risk of CVD after adjusting for smoking and hypertension (Inouye et al., 2018). Risk of depression is increased after acute myocardial infarction (Smolderen et al., 2017, 2009). It is also a marker of poor prognosis for myocardial infarction (Barefoot and Schroll, 1996). Therefore, these conditions are likely to share risk factors and pathophysiologic mechanisms (Hiles et al., 2015; Khandaker et al., 2019). Genome-wide association studies and epidemiological studies have reported relatively small genetic correlation between depression and CVD (Howard et al., 2019; Kendler et al., 2009; Khandaker et al., 2019), suggesting that shared environmental risk factors are key to this comorbidity.

Despite a large number of studies testing the links CVD and depression in adults, the association between CVD risk factors and depression in young people remains poorly understood. Existing studies of CVD risk factors and depression in young people have often focused on individual risk factors. Current/past smoking has been linked with increased risk of depression in longitudinal studies of adolescents (Choi et al., 1997; Duncan and Rees, 2005; Steuber and Danner, 2006). Longitudinal studies have also reported an association between high body mass index (BMI) and subsequent depression in both girls and boys (Frisco et al., 2013; Gomes et al., 2019; Monshouwer et al., 2012; Zhang et al., 2018). One study reported association between systolic blood pressure (SBP) and depression only in children/adolescents with parental history of depression (Hammerton et al., 2013). No obvious link between total cholesterol and depression has been found in adolescents (Waloszek et al., 2015). However, studies of other CVD risk factors, such as high-density lipoprotein (HDL), low-density lipoprotein (LDL) and triglycerides, and depression in young people are lacking.

While the concept of a composite CVD risk score for adults is well established in clinical practice (Hippisley-Cox et al., 2017), to our knowledge, no study has considered a variety of CVD risk factors in adolescents to determine their combined effect on subsequent mental health outcomes in young people. Rarer still are studies testing the direction of association between CVD risk and depression in young people. This work is important as this may provide clues regarding the origin of the comorbidity between CVD and depression. Studies based on young people are particularly advantageous as this age group is relatively less affected by confounders commonly present in older people, such as physical multi-morbidity.

Childhood determinants of adult CVD risk include a range of physical and social factors. The International Childhood Cardiovascular Cohort (i3C) Consortium was established to identify early-life risk factors associated with ideal cardiovascular health as defined by the American Heart Association (AHA) (Dwyer et al., 2013). A study of three i3C cohorts explored the association of the following factors with adult CVD risk: sex, age, ethnicity, family socioeconomic status, smoking, smoking, diet, BMI, SBP, HDL, LDL, and triglycerides (Laitinen et al., 2013). This study reported that family socioeconomic status and smoking in childhood were independently associated with adult cardiovascular health (Laitinen et al., 2013). Socioeconomic status and smoking are key indicators of social factors related to long term health outcomes, so called social determinants of health, which have been linked to depression in other studies (Assari, 2017).

In addition to social factors, which are important determinants of physical and mental health, biological processes could represent important shared mechanisms for CVD risk and depression. Inflammation could be one such mechanism, which is associated with both depression and CVD in adults. Using Mendelian randomisation analysis of data from the UK Biobank cohort, we recently reported that inflammation and triglycerides could be shared risk factors for depression and coronary heart disease (Khandaker et al., 2019). Circulating markers of inflammation such as interleukin-6 (IL-6) and C-reactive protein (CRP) are associated with depression and CVD in adults (Danesh et al., 2008, 2004; Halaris, 2017; Khandaker et al., 2014). Demonstrating an association between systemic inflammatory markers in childhood and subsequent CVD risk in young people would support the idea that childhood inflammation could be a shared mechanism for CVD and depression, but such studies are scarce.

**Fig. 1:**
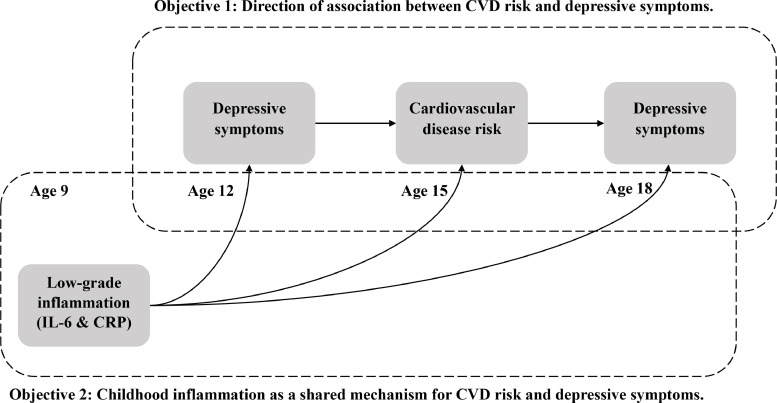
Conceptual model and objective for the analyses presented.

Using prospective data from the Avon Longitudinal Study of Parents and Children (ALSPAC) (Boyd et al., 2013), a general population birth cohort from the United Kingdom, we have investigated the directionality and potential mechanism of association between a range of CVD risk factors and depression in young people. Please see Fig. 1 for an overview of the analyses presented. Regarding direction of association, we have tested: (1) association between CVD risk score at age 15 and depressive symptoms at age 18; and (2) association between depressive symptoms at age 12 and CVD risk score at age 15. We hypothesised that similar to adults there will be evidence for bidirectional association between CVD risk score and depression in young people. Regarding mechanism of association, we have tested associations of IL-6 and CRP levels at age 9 with depressive symptoms at age 12 and with CVD risk score at age 15. In addition, we tested mediating effect of CVD risk score at age 15 on the association between inflammatory markers at age 9 and depressive symptoms at age 18. In line with the idea that inflammation could be a shared mechanism for depression and CVD, for these analyses, we hypothesised that early-childhood inflammatory markers would be associated with CVD risk score subsequently in adolescence, and that inflammatory markers at age 9 increase depression risk at age 18 by influencing CVD risk score at age 15.

## 2 Methods

### 2.1 Description of cohort and sample

ALSPAC is a general population-based birth cohort in the former Avon County in the South West region of England. Initially 14,541 pregnant women resident in the study catchment areas and with expected delivery dates between 1st April 1991 and 31st December 1992 were recruited into the cohort. Detailed information about the ALSPAC cohort can be found on the study website (http://www.bristol.ac.uk/alspac), and the sample characteristics and methodology have been previously described (Boyd et al., 2013; Fraser et al., 2013). For information on all available ALSPAC data, a fully searchable data dictionary is also available (http://www.bris.ac.uk/alspac/researchers/our-data).

Ethical approval for the study was obtained from ALSPAC Ethics and Law Committee and the Local Research Ethics Committees. Written informed consent was provided by all participants and no financial compensation was given.

The primary risk set for this study comprised 5007 unrelated individuals with CVD risk scores computed after imputation of missing data for individual risk factors (Supplementary Figure 1). Of the risk set, 3462 participants completed assessments at age 18 for depressive symptoms. We repeated the analyses based on 1810 participants with complete original data on all CVD risk factors.

### 2.2 Assessment of CVD risk factors at age 15

CVD risk factors were selected based on the AHA criteria for ideal cardiovascular health (Lloyd-Jones et al., 2010). The CVD risk score included: age, ethnicity, maternal social status, maternal smoking, own smoking, physical activity, BMI, SBP, LDL, HDL, and triglycerides. All variables were measured at age 15, except ethnicity, maternal smoking, and maternal social status which were assessed at birth.

Age (years) was used as a continuous variable. Self-reported ethnicity was originally coded as White, Black African, Black Caribbean, Black Other, Bangladeshi, Chinese, Indian, Pakistani, and Other. We recoded ethnicity as a binary variable (0=White; 1=any other ethnicity) due to low counts for ethnicities other than White. Self-reported maternal social status was documented using Office of National Statistics categories (Office for National Statistics, 2016). We recoded maternal social status as a binary variable (0=non-manual [I, II or IIIa]; 1=manual [IIIb, IV or V]). Members of the armed forces (*N* = 4) were excluded because this category was so small.

Mothers self-reported frequency of smoking during the first three months of pregnancy and again during the last two months of pregnancy (no cigarettes/day; 1–4 cigarettes/day; 5–9 cigarettes/day; 10–14 cigarettes/day; 5–19 cigarettes/day; 20–24 cigarettes/day; 25–29 cigarettes/day; more than 30 cigarettes/day). We used maternal smoking as a binary variable (0=non-smoker at both time points; 1=smoker at one or both time points). Own smoking was self-reported during the child-completed questionnaire and coded as an ordered categorical variable (no cigarettes/day; 1–5 cigarettes/day; 6–10 cigarettes/day; more than 10 cigarettes/day). We used own smoking as a binary variable (0=non-smokers; 1=smokers).

Participants self-reported frequency of physical activity during the past year (5 or more times per week; 1–4 times per week; 1–3 times a month; less than once a month). We created a binary measure of weekly exercise for analysis (0=exercise at least once per week; 1=exercise less than weekly).

We used BMI, SBP, HDL, LDL, and triglycerides as standardised continuous variables. To calculate BMI, weight was measured on Tania scales and height was measured using a Harpenden stadiometer. SBP was taken twice using a Dinamap 9301 Vital Signs Monitor (Morton Medical, London) and the mean of the two measurements was used in analysis. HDL, LDL and triglyceride concentrations were measured from fasting blood test as previously described (Lawlor et al., 2010).

### 2.3 Assessment of depressive symptoms at age 12

Depressive symptoms were self-reported using the Short Mood and Feelings Questionnaire (SMFQ) (Sharp et al., 2006) at age 12. The SMFQ is an age-appropriate, widely used and validated tool. It comprises 13 items covering core symptoms of depression and anxiety experienced in the past two weeks. Each item is scored zero (not true), one (sometimes true) or two (true) giving a total score of 0 to 26. Depressive symptoms score was used as a standardised continuous variable.

### 2.4 Assessment of depressive symptoms at age 18

Depressive symptoms were measured using a self-administered computerised version of the Clinical Interview Schedule Revised (CIS-R) (Lewis, 1994; Lewis et al., 1992). The CIS-R is a widely used, fully-structured assessment for measuring depression in large community samples (Lewis et al., 1992). The CIS-R includes questions about a range of symptoms including depression, depressive thoughts, fatigue, concentration, and sleep problems. CIS-R sums these symptoms scores to provide a total depressive symptoms score (0 to 21), reflecting the severity of depressive symptoms in the past week. Depressive symptoms score was used as a standardised continuous variable.

CIS-R also provides diagnosis of depressive episode (mild, moderate, or severe) according to International Classification of Disease 10th Revision (ICD-10) criteria. Depression diagnosis was used for presentation purposes in Table 1 only.

**Table 1:** Characteristics of the participants prior to imputation and using maximum available sample for each variable.

Characteristics *	All participants** (*N* = 5007)	Without depression at age 18 † (*N* = 3208)	With depression at age 18 † (*N* = 254)	Difference between individuals with and without depression at age 18 (T-test / Chi-square *p*-value)
**CVD risk factors ‡**				
CVD risk score – mean (SD)	0.0 (1.0)	−0.03 (1.0)	−0.01 (1.0)	0.84
Age (years) – mean (SD)	15.5 (0.3)	15.5 (0.3)	15.5 (0.3)	0.39
BMI (kg/m^2^) – mean (SD)	21.4 (3.5)	21.3 (3.4)	21.8 (3.7)	0.03
SBP (mmHg) – mean (SD)	122.9 (10.9)	123.1 (10.7)	121.2 (11.0)	0.01
LDL (mmol/L) – mean (SD)	2.1 (0.6)	2.1 (0.5)	2.1 (0.6)	0.11
HDL (mmol/L) – mean (SD)	1.3 (0.3)	1.3 (0.3)	1.3 (0.3)	0.89
Triglycerides (mmol/L) – mean (SD)	0.8 (0.4)	0.8 (0.4)	0.8 (0.3)	0.82
Ethnicity – no. White (%)	4763 (98.1)	3062 (97.9)	243 (98.4)	1.00
Maternal social status – no. manual (%)	642 (15.1)	404 (14.5)	33 (15.6)	0.91
Maternal smoking – no. smokers (%)	751 (15.9)	428 (14.0)	56 (23.7)	<0.001
Own smoking – no. smokers (%)	409 (11.8)	250 (9.8)	43 (22.3)	<0.001
Physical activity – no. less than weekly exercise (%)	792 (23.2)	551 (22.0)	50 (26.6)	0.02
**Potential confounders**				
Birthweight (kg) – mean (SD)	3.4 (0.5)	3.4 (0.5)	3.4 (0.6)	0.66
Total difficulties score (SDQ) at age 7 – mean (SD)	7.3 (4.7)	7.1 (4.5)	7.9 (5.1)	0.01
Maternal education – no. with less than O-level (%)	939 (19.3)	536 (17.1)	44 (17.7)	0.09
Sex – no. female (%)	2639 (52.7)	1725 (53.8)	189 (74.4)	<0.001
Family history of CVD – no. (%)	1080 (29.9)	902 (29.3)	80 (32.4)	0.16
**Inflammatory markers**				
IL-6 at age 9 (pg/mL) – mean (SD)	1.3 (1.6)	1.3 (1.5)	1.3 (1.4)	0.87
CRP at age 9 (mg/L) – mean (SD)	0.8 (2.7)	0.7 (1.9)	1.0 (4.3)	0.38
**Depression measures**				
Depressive symptoms (SMFQ) at age 12 – mean (SD)	4.0 (3.8)	3.8 (3.7)	5.9 (4.9)	<0.001
Depressive symptoms (CIS-R) at age 18 – mean (SD)	3.1 (3.9)	2.4 (2.9)	12.1 (3.3)	<0.001

* BMI: body mass index; CIS-R: Clinical Interview Schedule-Revised; CRP: C-reactive protein; CVD: cardiovascular disease; DAWBA: Development and Wellbeing Assessment; HDL: high density lipoprotein; IL-6: interleukin-6; LDL: low density lipoprotein; SBP: systolic blood pressure; SD: standard deviation; SDQ: Strengths and Difficulties Questionnaire; SMFQ: Short Mood and Feelings Questionnaire.

** Complete data for a single variable is 5007 participants, however many variables have missing data. For example, 4855 participants have data on ethnicity (4763 participants of White ethnicity is 98.1% of 4855).

† Depression is defined as ICD-10 mild/moderate/severe depression diagnosis at age 18. A total of 3462 participants have data on ICD-10 depression.

‡All CVD risk factors were assessed at age 15 except ethnicity, maternal smoking, and maternal social status which were assessed at birth.

### 2.5 Assessment of inflammatory markers at age 9

IL-6 and CRP were assayed from the blood samples collected from non-fasting participants. The blood samples were spun and frozen at −80 °C. After a median of 7.5 years in storage with no previous freeze-thaw cycles, IL-6 and CRP levels were measured by enzyme-linked immunosorbent assay (R&D Systems), and automated particle-enhanced immunoturbidimetric assay (Roche) respectively. All inter-assay coefficients of variation were less than 5% (Khandaker et al., 2014). We log-transformed IL-6 and CRP variables for analysis.

### 2.6 Assessment of potential confounders

Using the modified disjunctive cause criteria (VanderWeele, 2019), we selected the following confounders for adjustment: sex, birthweight, maternal education, Strengths and Difficulties Questionnaire (SDQ) total difficulties score at age 7, and family history of CVD. Variables in the CVD risk score were not included as confounders to prevent over-adjustment. Sex and birthweight were assessed at birth. Sex was coded as a binary variable. Birthweight (grams) was extracted from routine hospital birth records. Maternal education was self-reported at 32 weeks gestation as a categorical variable reflecting highest educational attainment (CSE/none; vocational; O-level; A-level; university degree). Mothers completed the parental version of the SDQ when their child was age 7. The SDQ is an age-appropriate, valid, and reliable tool for measuring psychological and behavioural problems in young children (Goodman, 1997). It measures problems in four domains: emotional, conduct, hyperactivity, and social/peer group. We used total difficulties score (0 to 40) as a standardised continuous variable. Family history of CVD (hypertension, diabetes, high cholesterol, or vascular disease) was self-reported by participants at the age 18 clinic assessment as a binary variable.

### 2.7 Statistical analysis

All analyses were carried out using R version 3.6.1.

### 2.8 CVD risk score at age 15

#### 2.8.1 Imputation of CVD risk factors

At least one CVD risk factor was available for 5007 participants but complete CVD risk scores were available for only 1810 participants. Analyses were conducted on complete cases after imputation of missing data for CVD risk variables (ethnicity, maternal social status, maternal smoking, own smoking, physical activity, BMI, SBP, LDL, HDL, and triglycerides). Age had no missing data. The percentage of missing data across the CVD risk factors varied between 1.7% and 36.4% (Supplementary Table 1).

We used the fully conditional Markov chain Monte Carlo method for multiple imputation of the CVD risk variables. We included auxiliary variables that were indicators of missingness (financial difficulties, life events, family income, and housing/living conditions) as well as confounder and outcome variables.

We used the “mice” package version 3.0 to create and analyse the multiply imputed datasets (van Buuren and Groothuis-Oudshoorn, 2011). Missing data were present in 20.1% of participants, so we used 20 imputations as recommended (White et al., 2011). We used predictive mean matching with 10 donors and type one matching. We separately estimated the parameters of interest in each dataset before combining using Rubin's rules.

#### 2.8.2 Calculation of CVD risk score at age 15

CVD risk factors were weighted based on their reported association with AHA ideal cardiovascular health from three i3C cohorts (Supplementary Table 2) (Dwyer et al., 2013; Laitinen et al., 2013). We summed the weighted CVD risk factors and applied z-transformation to create a standardised CVD risk score.

Seven i3C longitudinal cohort studies examine CVD risk factors in younger adults (Dwyer et al., 2013). The cohorts present beta estimates reflecting the association between CVD risk factors and AHA ideal cardiovascular health (Lloyd-Jones et al., 2010). One study combined three of these cohorts (Young Finns study, Childhood Determinants of Adult Health study, and the Princeton Follow-Up study) to give beta estimates for specific risk factors, adjusted for all other risk factors (Laitinen et al., 2013). Physical activity was adjusted for sex and age only. We used the relevant beta estimate(s) from this study as weights for our CVD risk factors (Supplementary Table 2). We used fixed effects meta-analysis as appropriate to combine multiple beta estimates.

We used BMI instead of waist circumference to match i3C cohort measurements (Supplementary Figure 2). Sex and family history of CVD were excluded from the CVD risk score because they independently produced distinct binomial distributions when they were included. Female sex is strongly associated with worse mental health outcomes while male sex is associated with increased CVD risk score. Fruit and vegetable consumption was also measured in the i3C Consortium but was not included in our CVD risk score due to the complexity of ALSPAC's diet-related variables.

#### 2.8.3 Direction of association between CVD risk and depressive symptoms

We used linear regression to assess the association between depressive symptoms at age 12 and CVD risk at age 15. Regression models were adjusted for sex, birthweight, maternal education, and family history of CVD.

We used linear regression to assess the association between CVD risk at age 15 (composite risk score and individual risk factors) and depressive symptoms at age 18. Regression models were adjusted for sex, birthweight, maternal education, and total difficulties score at age 7.

For continuous exposures, the beta estimates represent change in outcome (in SD) per SD increase in exposure. For binary exposures, the beta estimates represent change in outcome (in SD) for presence of risk factor compared with its absence.

### 2.9 Mechanism of association between CVD risk and depressive symptoms

#### 2.9.1 Association of inflammatory markers at age 9 with depressive symptoms at age 12

Regression models were estimated before and after adjustment for sex, birthweight, maternal education, and total difficulties score at age 7. We used linear regression to assess the association between IL-6/CRP concentration at age 9 and depressive symptoms at age 12. The beta estimates represent change in depressive symptoms (in SD) per unit increase in log-transformed IL-6/CRP values.

#### 2.9.2 Association of inflammatory markers at age 9 with CVD risk score at age 15

Regression models were estimated before and after adjustment for sex, birthweight, maternal education, and family history of CVD. We used linear regression to assess the association between IL-6/CRP concentration at age 9 and CVD risk score at age 15. The beta estimates represent change in CVD risk score (in SD) per unit increase in log-transformed IL-6/CRP values.

#### 2.9.3 Mediation analysis testing mediating effects of CVD risk score at age 15 on the association between inflammatory markers at age 9 and depressive symptoms at age 18

We conducted mediation analysis to test whether CVD risk score at age 15 mediates the relationship between IL-6/CRP at age 9 and depressive symptoms at age 18. Mediation models were computed before and after adjustments for sex, birthweight, maternal education, and total difficulties score at age 7. We analysed mediation within a path analysis framework using the “lavaan” R package (Rosseel, 2012). Lavaan uses full information maximum likelihood procedures to handle missing data (Rosseel, 2012). Non-parametric bootstrapping, based on 1000 bootstrap replicates, was used to calculate standard errors.

## 3 Results

### 3.1 Characteristics of the sample

Individuals meeting the ICD-10 criteria for depression at age 18, compared with those without depression, were more likely be female, smokers, have mothers who smoked, higher SDQ total difficulties score at age 7, and have higher BMI and lower SBP at age 18 (Table 1).

### 3.2 CVD risk score at age 15

Females had slightly higher CVD risk scores than males (Table 1 & Supplementary Figure 3). In the complete case set, the mean CVD risk score for females was 0.02 (SD=1.04) and for males was −0.02 (SD=0.95). After imputation (*N* = 5007), the mean CVD risk score for females was 0.03 (SD=1.04) and for males was −0.03 (SD=0.95).

### 3.3 Direction of association between CVD risk and depressive symptoms

#### 3.3.1 Association between depressive symptoms at age 12 and CVD risk score at age 15

In the total sample (*N* = 3226), depressive symptoms at age 12 were not associated with CVD risk score at age 15 (beta=0.03; SE=0.02; *p* = 0.09) (Table 2). Depressive symptoms at age 12 were also not associated with CVD risk score in sex-stratified analyses.

**Table 2:** Beta estimates (SE) for the association between cardiovascular disease risk score at age 15 and depressive symptoms at ages 12 and 18.

Model *	Participants	Sample (no.)	Unadjusted		Adjusted ‡	
			Beta (SE)	*p*-value	Beta (SE)	*p-value*
DEP at age 12 → CVD at age 15	All	3226	0.03 (0.02)	0.09	0.03 (0.02)	0.11
	Female	1760	0.03 (0.02)	0.21	0.03 (0.02)	0.18
	Male	1466	0.03 (0.03)	0.35	0.03 (0.03)	0.34
CVD at age 15 → DEP at age 18	All	3014	0.07 (0.02)	<0.001	0.06 (0.02)	<0.001
	Female	1647	0.09 (0.02)	<0.001	0.08 (0.02)	<0.001
	Male	1367	0.03 (0.02)	0.14	0.03 (0.02)	0.21

* CVD: cardiovascular disease risk score; DEP: depressive symptoms (measured using SMFQ at age 12 and CIS-R at age 18); SE: standard error.

‡ Adjusted for sex (if applicable), birthweight, maternal education, and SDQ total difficulties score at age 7/family history of CVD as appropriate.

#### 3.3.2 Association between CVD risk score at age 15 and depressive symptoms at age 18

In the total sample (*N* = 3014), CVD risk score at age 15 was associated with depressive symptoms score at age 18 (beta=0.07; SE=0.02; *p*<0.001) (Table 2). Evidence for this association remained after adjusting for confounders (adjusted beta=0.06; SE=0.02; *p*<0.001).

In sex-stratified analysis, CVD risk score was associated with depressive symptoms at age 18 in females (beta=0.09; SE=0.02; *p*<0.001) but not in males (beta=0.03; SE=0.02; *p* = 0.14). In females, evidence for association remained after adjusting for confounders (adjusted beta=0.08; SE=0.02; *p*<0.001).

#### 3.3.3 Association between individual CVD risk factors at age 15 and depressive symptoms at age 18

In the total sample (*N* = 3014), depressive symptoms at age 18 were associated with the following CVD risk factors at age 15: own smoking (beta=0.44; SE=0.05; *p*<0.001); maternal smoking (beta=0.20; SE=0.04; *p*<0.001); physical activity (beta=0.13; SE=0.04; *p*<0.001); maternal social status (beta=0.11; SE=0.05; *p* = 0.03); high LDL (beta=0.06; SE=0.02; *p*<0.001); and high BMI (beta=0.05; SE=0.01; *p*<0.01) (Table 3). After adjusting for confounders, evidence for association remained for own smoking and maternal smoking only.

**Table 3:** Beta (SE) for the association between individual cardiovascular disease risk factors at age 15 and depressive symptoms at age 18.

CVD risk factor * †	All participants (*N* = 3014)				Females (*N* = 1647)				Males (*N* = 1367)			
	Unadjusted		Adjusted ‡		Unadjusted		Adjusted ‡		Unadjusted		Adjusted ‡	
	Beta (SE)	*p-value*	Beta (SE)	*p-value*	Beta (SE)	*p-value*	Beta (SE)	*p-value*	Beta (SE)	*p-value*	Beta (SE)	*p-value*
Own smoking (no vs yes)	0.44 (0.05)	<0.001	0.39 (0.05)	<0.001	0.47 (0.07)	<0.001	0.44 (0.07)	<0.001	0.30 (0.08)	<0.001	0.29 (0.08)	<0.001
Maternal smoking (no vs yes)	0.20 (0.04)	<0.001	0.17 (0.04)	<0.001	0.32 (0.06)	<0.001	0.26 (0.06)	<0.001	0.06 (0.06)	0.27	0.06 (0.06)	0.29
Physical activity (weekly vs less than weekly)	0.13 (0.04)	<0.001	0.06 (0.04)	0.16	0.07 (0.05)	0.19	0.04 (0.05)	0.41	0.09 (0.06)	0.15	0.08 (0.06)	0.19
Maternal social status (non-manual vs manual)	0.11 (0.05)	0.03	0.06 (0.05)	0.23	0.08 (0.06)	0.24	0.03 (0.07)	0.68	0.10 (0.06)	0.11	0.10 (0.06)	0.12
Ethnicity (White vs any other ethnicity)	−0.08 (0.11)	0.45	−0.11 (0.11)	0.33	−0.15 (0.16)	0.35	−0.17 (0.16)	0.27	−0.01 (0.15)	0.92	−0.02 (0.15)	0.92
LDL (mmol/L)	0.06 (0.02)	<0.001	0.03 (0.02)	0.09	0.03 (0.03)	0.28	0.02 (0.03)	0.38	0.04 (0.02)	0.65	0.04 (0.02)	0.08
BMI (kg/m^2^)	0.05 (0.02)	<0.01	0.03 (0.02)	0.08	0.06 (0.02)	<0.01	0.05 (0.02)	0.02	−0.01 (0.02)	0.68	−0.01 (0.02)	0.54
Triglycerides (mmol/L)	0.01 (0.02)	0.57	<0.01 (0.02)	0.90	0.01 (0.03)	0.81	<0.01 (0.03)	0.98	<0.01 (0.02)	0.99	−0.01 (0.02)	0.81
SBP (mmHg)	−0.03 (0.02)	0.03	<0.01 (0.02)	0.90	<0.01 (0.02)	0.93	−0.01 (0.02)	0.80	0.01 (0.02)	0.69	0.01 (0.02)	0.71
HDL (mmol/L)	−0.01 (0.02)	0.76	−0.05 (0.02)	0.03	−0.05 (0.03)	0.05	−0.05 (0.03)	0.08	−0.04 (0.03)	0.10	−0.04 (0.03)	0.12
Age (years)	−0.01 (0.02)	0.57	−0.02 (0.02)	0.21	<0.01 (0.03)	0.92	<0.01 (0.03)	0.88	−0.05 (0.03)	0.08	−0.05 (0.03)	0.05

* BMI: body mass index; CVD: cardiovascular disease; HDL: high density lipoprotein; LDL: low density lipoprotein; SBP: blood pressure; SE: standard error.

† Own smoking, maternal smoking, physical activity, maternal social status, and ethnicity were used as binary variables. LDL, BMI, triglycerides, SBP, HDL, and age were used as standardised continuous variables (per 1 SD increase in exposure). The outcome for all analyses was standardised depressive symptoms score (1 SD increase in outcome).

‡ Adjusted for sex (if applicable), birthweight, maternal education, and SDQ total difficulties score at age 7.

In sex-stratified analysis, own smoking, maternal smoking, high BMI, and low HDL were associated with depressive symptoms in females (Table 3). Evidence for association remained for own smoking, maternal smoking, and BMI after adjusting for confounders. In males, own smoking was associated with depressive symptoms. Evidence for association remained after adjusting for confounders.

### 3.4 Mechanism of association between CVD risk and depressive symptoms

#### 3.4.1 Associations between inflammatory markers at age 9 and depressive symptoms at age 12

In the total sample (*N* = 2574), IL-6 concentration at age 9 was associated with depressive symptoms at age 12 (beta=0.13; SE=0.05; *p*<0.01) (Table 4). Evidence for association did not remain after adjusting for confounders. In the total sample (*N* = 2574), CRP concentration at age 9 was not associated with depressive symptoms at age 12 (beta=0.06; SE=0.04; *p* = 0.12).

**Table 4:** Beta estimate (SE) for the associations of IL-6 and CRP levels at age 9 with depressive symptoms at age 12 and with cardiovascular disease risk score at age 15.

Model *	Participants	Sample (no.)	Unadjusted		Adjusted ‡	
			Beta (SE)	*p-value*	Beta (SE)	*p-value*
IL-6 at age 9 → DEP at age 12	All	2574	0.13 (0.05)	<0.01	0.08 (0.05)	0.11
	Female	1303	0.17 (0.08)	0.03	0.15 (0.08)	0.06
	Male	1271	0.03 (0.07)	0.62	0.02 (0.07)	0.76
CRP at age 9 → DEP at age 12	All	2574	0.06 (0.04)	0.12	0.01 (0.04)	0.79
	Female	1303	0.08 (0.06)	0.15	0.07 (0.06)	0.21
	Male	1271	−0.04 (0.05)	0.44	−0.05 (0.05)	0.31
IL-6 at age 9 → CVD at age 15	All	2282	0.32 (0.05)	<0.001	0.30 (0.05)	<0.001
	Female	1244	0.42 (0.08)	<0.001	0.40 (0.08)	<0.001
	Male	1038	0.20 (0.08)	<0.01	0.21 (0.07)	<0.01
CRP at age 9 → CVD at age 15	All	2282	0.40 (0.04)	<0.001	0.39 (0.04)	<0.001
	Female	1244	0.42 (0.06)	<0.001	0.41 (0.06)	<0.001
	Male	1038	0.37 (0.06)	<0.001	0.36 (0.06)	<0.001

* CRP: C-reactive protein; CVD: cardiovascular disease risk score; DEP: depressive symptoms (measured using SMFQ at age 12 and CIS-R at age 18); IL-6: interleukin-6; SE: standard error.

‡ Adjusted for sex (if applicable), birthweight, maternal education, and SDQ total difficulties score at age 7/family history of CVD as appropriate.

#### 3.4.2 Associations between inflammatory markers at age 9 and CVD risk at age 15

In the total sample (*N* = 2282), IL-6 concentration at age 9 was associated with CVD risk score at age 15 (beta=0.32; SE=0.05; *p*<0.001) (Table 4). In the total sample (*N* = 2282), CRP concentration at age 9 was also associated with CVD risk score at age 15 (beta=0.40; SE=0.04; *p*<0.001). Evidence for these associations remained after adjusting for confounders.

#### 3.4.3 Mediating effect of CVD risk at age 15 on the association between inflammatory markers at age 9 and depressive symptoms at age 18

In the total sample (*N* = 2004), there was evidence that CVD risk score at age 15 mediated the association of depressive symptoms at age 18 with both IL-6 (indirect effect: beta=0.02; SE=0.01; *p* = 0.01) and CRP concentrations at age 9 (indirect effect: beta=0.02; SE=0.01; *p*<0.01) (Table 5). Evidence for these associations remained after adjusting for potential confounders.

**Table 5:** Mediating effects of CVD risk score at age 15 on the association between IL-6/CRP at age 9 and depressive symptoms at age 18.

Model *	Direct/indirect effect	Unadjusted (*N* = 2004)		Adjusted (*N* = 2004) ‡	
		Beta (SE)	*p-value*	Beta (SE)	*p-value*
IL-6 at age 9 → CVD at age 15 → DEP at age 18	Direct effect	0.11 (0.05)	0.02	0.04 (0.05)	0.34
	Indirect effect	0.02 (0.01)	0.01	0.02 (0.01)	0.02
	Total effect	0.14 (0.05)	<0.01	0.06 (0.05)	0.19
CRP at age 9 → CVD at age 15 → DEP at age 18	Direct effect	<0.01 (0.04)	0.90	−0.06 (0.03)	0.07
	Indirect effect	0.02 (0.01)	<0.01	0.02 (0.01)	<0.01
	Total effect	0.03 (0.03)	0.40	−0.04 (0.03)	0.25

* CRP: C-reactive protein; CVD: cardiovascular disease risk score; DEP: depressive symptoms (measured using CIS-R); IL-6: interleukin-6.

‡ Adjusted for sex (if applicable), birthweight, maternal education, and SDQ total difficulties score at age 7.

### 3.5 Sensitivity analysis

We repeated analyses testing the direction of association between CVD risk and depressive symptoms based on the complete case set (*N* = 1810). The results were similar (Supplementary Table 3).

## 4 Discussion

This population-based longitudinal study in young people is one of the first to use a composite CVD risk score to investigate the associations between CVD risk and depressive symptoms in childhood, adolescence and early-adulthood. In particular, we tested the direction of association between CVD risk and depressive symptoms and the potential role of childhood inflammation. We report that higher CVD risk score in mid-adolescence at age 15 is associated with depressive symptoms subsequently in early-adulthood at age 18. However, depressive symptoms at age 12 were not associated with the CVD risk score at age 15. We also report that childhood IL-6 and CRP concentration at age 9 are associated with CVD risk score at age 15. Furthermore, IL-6/CRP concentrations at age 9 appear to influence the risk of depressive symptoms at age 18 by via CVD risk score at age 15.

### 4.1 *Direction of association between CVD risk and depressive symptoms*

The first objective of our analysis was to determine whether CVD risk and depressive symptoms are bidirectionally associated in young people. We report that higher CVD risk in mid-adolescence was associated with depressive symptoms in early-adulthood. Own smoking and maternal smoking were particularly strongly associated with depressive symptoms. BMI was associated with subsequent depressive symptoms in female participants only. BMI is a complex trait which could be influenced by a genetics and a variety of environmental factors. The lack of association between depressive symptoms in childhood and subsequent CVD risk in mid-adolescence may suggest a unidirectional relationship in young people, where high CVD risk predicts subsequent depressive symptoms but not the other way around. Our findings are consistent with longitudinal studies reporting that high BMI and smoking are associated with depressive symptoms in young people (Choi et al., 1997; Duncan and Rees, 2005; Frisco et al., 2013; Gomes et al., 2019). Other studies have reported an association between depressive symptoms in adolescence and subclinical measures of CVD that predict CVD in adulthood (Dietz and Matthews, 2011). Although we found no evidence of an association between childhood depressive symptoms at age 12 and subsequent CVD risk in adolescence at age 15 in our sample, it is possible that childhood depressive symptoms may have a cumulative effect on CVD risk in young people which is not yet evident by age 15.

Social factors are important determinants of CVD risk and depression in young people. Mother's educational attainment and socioeconomic status have been shown to influence the relationship between CVD risk and depression in childhood (Gilman et al., 2008; Najman et al., 1998). Consistent with this literature, we found some evidence for an association between maternal social status and depressive symptoms in early-adulthood. Low socioeconomic status is associated with maternal smoking (Gilman et al., 2008; Najman et al., 1998), and socioeconomic gradients in adiposity and blood pressure exist in children at age 10, suggesting that inequalities in CVD risk factors will widen over time, along with depression cases (Howe et al., 2010). Withdrawal symptoms from smoking are also likely to exert psychological effects including regular mood fluctuations, which may increase risk of developing depression (Berk et al., 2013; Munafò et al., 2008). In addition, a diet high in trans-fatty acids may contribute to the association between CVD risk and depression via increases in plasma LDL-cholesterol levels, proinflammatory cytokines, and endothelial dysfunction (Sanchez-Villegas and Martínez-González, 2013). Diverse psychosocial factors therefore influence the development of depression partly through CVD risk factors.

### 4.2 *Mechanism of association between CVD risk and depressive symptoms*

The second part of our analysis focused on childhood inflammation as a potential mechanism for the comorbidity between CVD risk and depression. We report that childhood IL-6 and CRP levels are associated with higher CVD risk score in mid-adolescence, and that adolescent CVD risk score mediates the association between childhood IL-6/CRP levels and depressive symptoms in early-adulthood. Infection and inflammation have been implicated in the pathogenesis of depression (Benros et al., 2013; Chaplin et al., 2020; Khandaker et al., 2014) and there is substantial evidence also linking inflammation with CVD risk (Welsh et al., 2017; Willerson and Ridker, 2004; Zhang et al., 2017). Childhood inflammation therefore could be a common risk factor for both depression and CVD. This idea is consistent with Barker's common cause hypothesis which suggests that exposure to risk factors during a critical developmental window may alter certain physiologic system(s) leading to increased risk of chronic illnesses subsequently in adulthood (Barker and Osmond, 1986). Our findings suggest that inflammation could be one such common pathophysiologic mechanism because IL-6/CRP levels at age 9 were associated with CVD risk score at age 15 in this study. A previous study from the same cohort reported that childhood IL-6 levels were associated with depressive symptoms at age 18 (Khandaker et al., 2014).

Inflammation may have different effects on different organs. In the brain, inflammation may lead to symptoms of depression, while inflammatory processes may contribute to changes in the cardiovascular system leading to increased risk of CVD. For instance, human and preclinical studies suggest that systemic inflammation may increase risk of depression by decreasing synaptic serotonin, and by increasing CNS levels of glutamate, excitotoxicity and oxidative stress (Dantzer et al., 2008; Miller and Raison, 2016). Similarly, inflammation has been reported to be associated with atherosclerosis (Libby, 2002) and endothelial dysfunction (Castellon and Bogdanova, 2016). Our findings are consistent with this literature suggesting that childhood inflammatory markers may increase the risk of depressive symptoms in early-adulthood by influencing adolescent CVD risk score.

Inflammation and CVD risk in young people may form a vicious cycle that perpetuates pathophysiologic changes and ultimately increase the risk of both CVD and depression in adults. For instance, inflammation is linked with insulin resistance (Andreas et al., 2003). On the other hand, certain CVD risk factors, such as obesity, can also increase inflammation. Obesity has been reported to be associated with increased secretion of inflammatory mediators and low-grade inflammation due to progressive accumulation of adipocytes, macrophages and T cells in white adipose tissue, muscles and the liver (Capuron et al., 2017). Overweight individuals also have increased gut permeability to bacteria and low bacterial diversity which further contribute to systemic inflammation (Capuron et al., 2017).

### 4.3 *Limitations*

Our analysis is not without limitations. First, we used BMI in the CVD risk score as a measure of central adiposity. BMI is not necessarily the most appropriate measure to use, particularly in adolescents. However, using waist circumference instead of BMI made no difference to the CVD risk score distribution. In addition, although the AHA cardiovascular health guidelines have been shown to be applicable to CVD risk in younger adults, data on diet were not available to us and were not included in our CVD risk score. Relatively few participants had complete data, reducing statistical power. We attempted to address this issue by multiple imputation for missing data. Our results appear to show a sex difference in the association between CVD risk and subsequent depressive symptoms, but this should be interpreted with caution in light of sample attrition and reduced statistical power. The potential sex difference may be related to the onset of puberty, which needs to be tested in future studies. Finally, the cohort is primarily composed of White individuals, limiting the generalisability of findings and the statistical power to adequately explore the effect of ethnicity.

## 5 *Conclusions*

The association between CVD risk and depressive symptoms in childhood/adolescence is unidirectional, with higher CVD risk increasing the risk of depressive symptoms. Childhood inflammation may increase risk of depression by influencing adolescent CVD risk. This model where inflammation is a shared, modifiable risk factor for adult CVD and depression requires replication in other samples, but may have important implications for the prevention of CVD and depression.

## 6 Funding

The UK Medical Research Council and Wellcome (Grant ref: 102,215/2/13/2) and the University of Bristol provide core support for ALSPAC. The publication is the work of the authors who will serve as guarantors for the contents of this paper. A comprehensive list of grants funding is available on the ALSPAC website (http://www.bristol.ac.uk/alspac/external/documents/grant-acknowledgements.pdf).

This report of research supported in part by the National Institute for Health Research (NIHR) Applied Research Collaboration (ARC) East of England expresses the views of the authors and not necessarily those of the NHS, the NIHR, the Department of Health and Social Care or other funding bodies.

ABC is supported by the NIHR CLAHRC RCF (MRR73-1795-00000) (https://www.nihr.ac.uk/), NIHR ARC East of England, the MQ: Transforming Mental Health (Data Science Award; grant code: MQDS17/40) (https://www.mqmentalhealth.org/home/), and Wolfson College (https://www.wolfson.cam.ac.uk/). GMK acknowledges funding support from the Wellcome Trust (grant code: 201486/Z/16/Z) (https://wellcome.org/), MQ as above, the Medical Research Council (grant code: MC_PC_17213 and MR/S037675/1) (https://mrc.ukri.org/), and the BMA Foundation (J Moulton grant 2019) (http://www.bmafoundationmr.org.uk/). PBJ acknowledges funding from MQ, NIHR ARC East of England and the Medical Research Council, as above, and from NIHR PGfAR 0616-20,003. The funders had no role in study design, data collection and analysis, decision to publish, or preparation of the manuscript.

## Declaration of Competing Interest

The authors have no conflict of interest to declare.

## Appendix Supplementary materials

Image, application 1
