# Supplementary material for: Direction of association between Cardiovascular risk and depressive symptoms during the first 18 years of life: A prospective birth cohort study
Source: J Affect Disord. 2021 Sep 1;292:508–16. doi: 10.1016/j.jad.2021.05.094 (PMC8324768; doi:10.1016/j.jad.2021.05.094)
Supplement: Supplementary file 1 [file mmc1.docx]

**Direction of Association between Cardiovascular Risk and Depressive Symptoms during the First 18 Years of Life: A Prospective Birth Cohort Study**

**Supplementary Materials**

**Page 2:** Supplementary Figure 1. Flow chart showing inclusion of study participants after imputation.

**Page 3:** Supplementary Figure 2. Distribution of cardiovascular disease (CVD) risk score including body mass index (BMI) or waist circumference (WC) under different conditions.

**Page 4:** Supplementary Figure 3. CVD risk score distribution at age 15 (A) after imputation, and (B) in complete case set.

**Page 5:** Supplementary Table 1. Missing data in the risk set (N=5007) before imputation.

**Page 6:** Supplementary Table 2. Beta coefficients used as weights in the cardiovascular disease risk score based on the association between risk factors from i3C Consortium cohorts and American Heart Association ideal cardiovascular health.

**Page 7:** Supplementary Table 3. Beta estimate (SE) for the association between cardiovascular disease risk score at age 15 and depressive symptoms at age 12/18 in the complete case set.

**Supplementary Figure 1. Flow chart showing inclusion of study participants after imputation.** The number in brackets represents the number of participants prior to imputation i.e. the complete case set. CRP: C-reactive protein; CVD: cardiovascular disease; IL6: interleukin-6

**Total sample**

**N = 15376**

**Total N for CVD risk score age 15 = 5007 (1810)**

**Total N confounders = 3014 (1390)**

**Total N for depressive symptoms age 12 = 4491 (1706)**

**Total N for depressive symptoms age 18 = 3462 (1504)**

**Total N for CRP/IL-6**

**age 9 = 3127 (1371)**

**Total N confounders = 3226 (1466)**

**Total N confounders = 2282 (1181)**

**Supplementary Figure 2. Distribution of cardiovascular disease (CVD) risk score including body mass index (BMI) or waist circumference (WC) under different conditions.** All CVD variables refers to age, ethnicity, maternal social status, maternal smoking, own smoking, physical activity, BMI/WC, systolic blood pressure, LDL, HDL, triglycerides, sex, and family history of CVD.


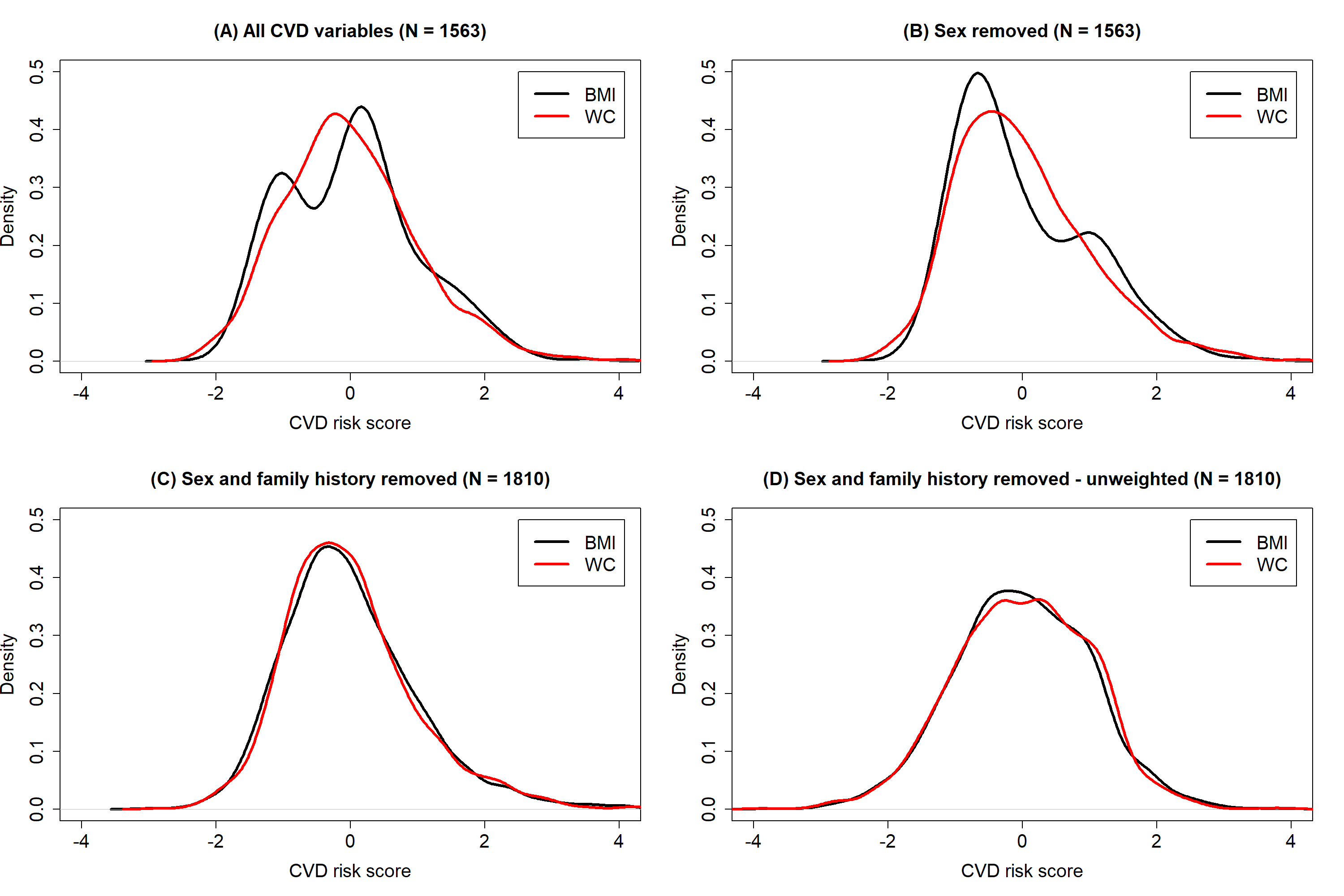


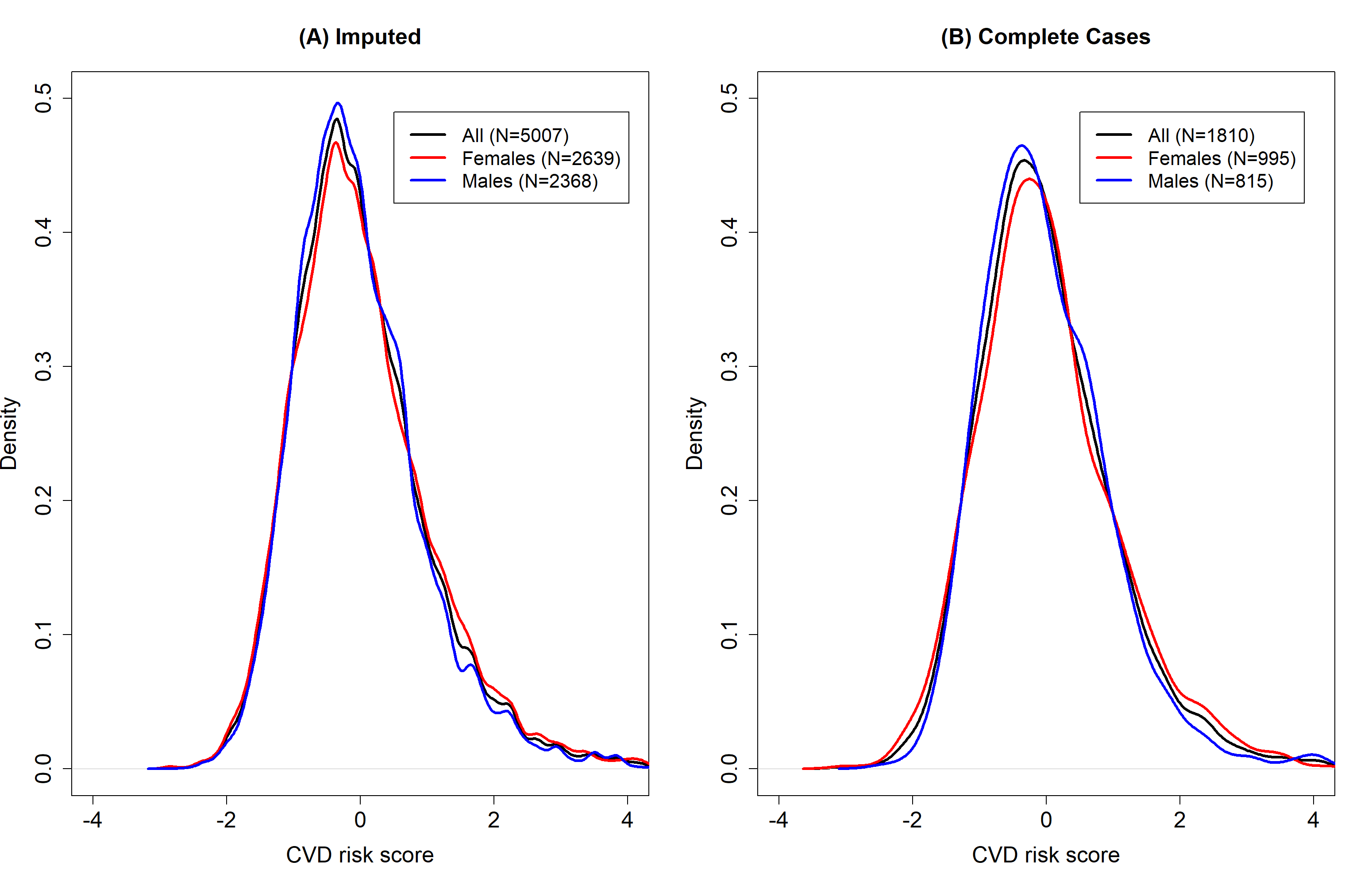
**Supplementary Figure 3. CVD risk score distribution at age 15 (A) after imputation, and (B) in complete case set.**

| Supplementary Table 1. Missing data in the risk set (N=5007) before imputation. | | |
| --- | --- | --- |
| CVD risk factor * | Participants with missing data (no.) | Percentage missing data (%) |
| Age (years) | 0 | 0 |
| BMI (kg/m^2^) | 84 | 1.7 |
| Ethnicity (White vs any other ethnicity) | 152 | 3.0 |
| SBP (mmHg) | 176 | 3.5 |
| Maternal smoking (no vs yes) | 294 | 5.9 |
| Maternal social status (non-manual vs manual) | 743 | 14.8 |
| Own smoking (no vs yes) | 1540 | 30.8 |
| Physical activity (weekly vs less than weekly) | 1588 | 31.7 |
| LDL (mmol/L) | 1823 | 36.4 |
| HDL (mmol/L) | 1823 | 36.4 |
| Triglycerides (mmol/L) | 1823 | 36.4 |
| Total missing data | - | 20.1 |
| * BMI: body mass index; CVD: cardiovascular disease; CI: confidence intervals; HDL: high density lipoprotein; LDL: low density lipoprotein; SBP: blood pressure. | | |

| **Supplementary Table 2. Beta coefficients used as weights in the cardiovascular disease risk score based on the association between risk factors from i3C Consortium cohorts and American Heart Association ideal cardiovascular health**. | | | | |
| --- | --- | --- | --- | --- |
| CVD risk factor * | Beta (p-value) | | | Beta coefficient used as weight in CVD risk score ǂ |
|  | YFS (N=1668) | CDAH (N=1365) | PFS (N=659) |  |
| Age (years) | -0.04 (0.67) | 0.06 (<0.01) | 0.03 (0.09) | 0.02 |
| Sex (female vs male) | -1.06 (0.07) | -0.99 (0.06) | -0.83 (0.09) | -0.98 |
| Ethnicity (White vs African American) † | - | - | -0.16 (0.18) | -0.16 |
| Socioeconomic status | 0.21 (<0.01) | 0.13 (<0.01) | 0.12 (<0.01) | 0.09 ⸙ |
| Parental smoking (no vs yes) | -0.26 (<0.01) | -0.10 (0.12) | - | -0.18 |
| Own smoking (no vs yes) | - | -0.38 (0.12) | - | -0.38 |
| BMI (kg/m^2^) | -0.08 (0.42) | -0.10 (<0.01) | -0.07 (<0.01) | -0.08 |
| SBP (per 10 mm/Hg) | -0.09 (<0.01) | - | - | -0.09 |
| LDL (mmol/l) | -0.25 (<0.01) | - | -0.10 (0.09) | -0.19 |
| HDL (mmol/l) | - | - | 0.27 (0.09) | 0.27 |
| Triglycerides (mmol/l) | -0.17 (0.16) | - | -0.09 (0.45) | -0.13 |
| Physical activity ‡ | 0.01 (0.81) | -0.01 (0.24) | - | -0.01 |
| * BMI: body mass index; BP: blood pressure; CDAH: Childhood Determinants of Adult Health study; CVD: cardiovascular disease; LDL: low density lipoprotein; HDL: high density lipoprotein; PFS: Princeton Follow-up Study; SBP: systolic blood pressure; YFS: Young Finns Study.  ǂ Fixed effects meta-analysis used as appropriate to combine multiple estimates.  ⸙ Rescaled coefficient to reflect ALSPAC measure of maternal socioeconomic status since family income data is not available in ALSPAC.  † Due to a lack of comparable published data from the UK, the i3C Consortium estimate for ethnicity (White vs African American) was interpreted as equivalent to White vs any other ethnicity.  ‡ Adjusted for age and sex only. YFS (N=1810) measured variable as physical activity index. CDAH (N=1417) measured variable as physical activity hours per week. | | | | |

| **Supplementary Table 3. Beta estimate (SE) for the association between cardiovascular disease risk score at age 15 and depressive symptoms at age 12/18 in the complete case set.** | | | | | | | |
| --- | --- | --- | --- | --- | --- | --- | --- |
| Model * | Participants | Unadjusted | |  | Adjusted ‡ | | |
|  |  | Sample (no.) | Beta (SE) | P-value | Sample (no.) | Beta (SE) | P-value |
| CVD at age 15 🡪 DEP at age 18 | All | 1504 | 0.02 (0.02) | 0.36 | 1390 | 0.01 (0.02) | 0.43 |
|  | Female | 832 | 0.02 (0.03) | 0.53 | 757 | 0.01 (0.03) | 0.65 |
|  | Male | 672 | 0.01 (0.03) | 0.64 | 633 | 0.01 (0.03) | 0.62 |
| DEP at age 12 🡪 CVD at age 15 | All | 1706 | 0.03 (0.02) | 0.23 | 1466 | 0.02 (0.03) | 0.52 |
|  | Female | 933 | 0.02 (0.03) | 0.48 | 804 | 0.02 (0.03) | 0.47 |
|  | Male | 733 | 0.03 (0.04) | 0.44 | 662 | <0.01 (0.04) | 0.98 |
| *CVD: cardiovascular disease risk score; DEP: depression measured using Short Mood and Feelings Questionnaire at age 12 and using Clinical Interview Schedule Revised at age 18; SE: standard error.  ‡ Adjusted for sex (if applicable), birthweight, maternal education, and SDQ total difficulties score at age 7/family history of CVD as appropriate. | | | | | | | |
